# Prevalence, Risk Factors and Outcomes Associated with Physical Restraint in Acute Medical Inpatients over 4 Years—A Retrospective Cohort Study

**DOI:** 10.3390/geriatrics8010015

**Published:** 2023-01-17

**Authors:** Umberto Spennato, Nathalie Lerjen, Jennifer Siegwart, Beat Mueller, Philipp Schuetz, Daniel Koch, Tristan Struja

**Affiliations:** 1 Department of Internal Medicine, Cantonal Hospital Aarau, Medical University Clinic, 5001 Aarau, Switzerland; 2 Medical Faculty Department of Clinical Research, University of Basel, 4056 Basel, Switzerland

**Keywords:** sensor mats, bedrails, belt, blanket restrictions, physical restraints, patients, confusion, delirious behavior, falls

## Abstract

Background: Physical restraints are frequently used in acute care hospitals. Their application is associated with negative outcomes, while their intended preventive effect is debated. Objectives: To determine the prevalence of physical restraints and associated outcomes on medical wards in a tertiary care hospital. Methods: Retrospective cohort study (January 2018 to December 2021). We included all adult medical in-patients and excluded patients with admission to the intensive care unit, short stays (length of stay (LOS) < 48 h), and patients declining informed consent. Results: Of 11,979 admissions, the prevalence of patients with at least one restraint was 6.4% (n = 772). Sensor mats were used most frequently (73.0%, n = 666), followed by blanket restrictions (14.5%, n = 132), bedrails (8.8%, n = 80) and belts (3.7%, n = 34). On average, restraints were applied 19 h (standard deviation (SD) ± 161) before a fall. Average restraint duration was 42 h (SD ± 57). Patients with a restraint had longer LOS 8 days (IQR 5–14) vs. 5 days (IQR 3–9). Median nurses’ time expenditure was 309 h (IQR 242–402) vs. 182 h (IQR 136–243) for non-restrained patients. Patients with restraints fell more often (22.5% vs. 2.7%) and were more likely to die (13.3% vs. 5.1%). These differences persisted after adjusting a regression model for important clinical confounders. We saw a decline in the duration of restraints over the years, but no variation between wards. Conclusion: Approximately 6% of medical patients, mostly older and severely ill, were affected by restraint use. For the first time, we report data over 4 years up to ward-level granularity.

## 1. Introduction

Physical restraints are defined as the application of devices (including belts, harnesses, manacles, sheets, and straps) nearby or to a person’s body to restrict their movement and to prevent the person from harming themselves or endangering others, or to ensure that essential treatment can be provided [1]. The efficacy of these methods has widely been discussed especially regarding their ethical, physical, and psychological implications and intended effect [2]. Additionally, their usage differs widely, making comparisons difficult.

While data on restraint use in general wards are scarce, there is some cross-country information on the use of restraints in the intensive care unit (ICU) setting available. The PRICE Study compared prevalence in ICUs across nine European countries with a total of 566 patients and found a large variation with prevalence ranging from 0.0% to 100%. However, these results need to be interpreted with caution because sample sizes differed between 15 and 319 patients [3]. Not surprisingly, Minnick et al. found that restraining rates on regular wards were much lower than in ICUs. Still, major differences were found between different clinics. For example, geriatric units had a much higher rate of restraint use than did medical, cardiac, or oncology units [4].

A cross-sectional study in a German acute care hospital found a prevalence rate of restraints of 11.8% (n = 1276). Then again, restraint use differed greatly ranging from 0 to 31% on general wards [5].

Even scarcer data are available on trends over time on restraint use. A study from Kwok et al. in China looked at 1946 patients admitted to medical wards in 2007 and 2009. With the implementation of a restraint reduction program, their use declined significantly from 13.3% in 2007 to 4.1% in 2009 [6]. Unfortunately, the authors did not provide specific information on the implemented program. 

The scope of this study is to determine the prevalence, the types, and duration of physical restraints in association with length of stay, nurse’s time expenditure hours, falls, and in-hospital death on general wards in an acute care hospital. We hypothesized that patients with physical restraints would have longer length of stay, higher nurse’s time expenditure hours, more falls, and a higher rate of in-hospital death compared to patients without restraint use. Furthermore, we describe the usage of restraints on different wards of the same clinic over time.

## 2. Methods

This retrospective cohort study was conducted at the Cantonal Hospital Aarau, a tertiary, 600-bed hospital in Switzerland. Our Medical University Clinic has roughly 6000 admissions per year of which 80% are emergency admissions. Indications for the use of physical restraint were based on internal standard operating procedures regulated by the Swiss Civil Law [7].

### 2.1. Inclusion and Exclusion Criteria

All emergency admissions from January 2018 to December 2021 were screened for eligibility. The inclusion criteria were age ≥18 years, and hospitalization on a medical ward. 

Exclusion criteria were non-medical leading diagnoses, length of hospital stay (LOS) <48 h and declined general informed consent. We deliberately excluded admission to an ICU, as these patients are very distinct from patients in a regular ward and have a very high rate of restraint use. We specifically looked at physical restraints, such as blanket restriction, bed rails, belts, and sensor mats. We excluded motion sensor alarms, as their use at our institution is experimental. It has to be noted that due to local regulations, regular, pre-installed and low-raised bed rails are not considered a physical restraint. Only additionally inserted high-raise bed rails are considered a physical restraint. Further, we excluded one-to-one supervision, as it is not a form of physical restraint by Swiss Civil Law.

### 2.2. Outcomes 

Primary outcomes were LOS, nurses’ time expenditure per case, fall and in-hospital death. Previous studies have shown that patients with restraints have much longer LOS, putting additional strain on personnel, and are more likely to suffer adverse in-hospital outcomes [6,8,9]. A secondary outcome was the use of restricting measures in patients with delirium due to the high risk of patients suffering a delirium to be restrained [10].

### 2.3. Data Collection

We used administrative data provided by the coding department as well as data from the electronic patient record, which includes only in-hospital outcomes. We extracted the number and duration of every physical restraint at hospital admission based on the actual restraining, which provides us with an accurate database as Swiss law mandates that every restraining order is registered in a patient’s health record. Further, we manually conducted quality checks on a random subsample of patients. Data on pharmacological treatment and reason for a particular indication of a physical restraint were obtained by hand searching electronic health records for patients with restraints only by two authors (NL and TS) analogous to a previous study [11]. Per internal protocol, nurses screened patients older than 65 years once per shift (i.e., three times per day) for the first three days of admission for the risk of delirium using the Delirium Observational Screening Scale (DOS) [12]. A DOS ≥ 3 would trigger the confusion assessment method short (CAM-short) assessment to ascertain the presence of delirium [13]. Nurses’ time expenditure includes both working time from registered nurses and their assistants. In our EHR, the time expenditure for common tasks performed by nurses on a daily basis is standardized (e.g., insertion of an intravenous line is credited with 15 min). Nurses document these tasks regularly during their day. In the case of more demanding patients, nurses can modify the standardized times. Although the performed tasks might not be documented eventually, our measure provides at least an average and conservative estimate.

### 2.4. Statistical Analysis

We used descriptive statistics including mean with standard deviation (SD), median with interquartile range (IQR), and frequencies to describe the population, as appropriate. A two-sided *p*-value < 0.05 was considered significant. To analyze restraint use over time, we first fitted a linear regression with the number of restraints as the dependent variable and the year as the sole independent variable. We then fitted a second model additionally adjusting for defined clinically relevant confounders and effect modifiers, such as age, Elixhauser comorbidity index, gender, main diagnosis, insurance class, and ward. For the continuous outcome LOS and nurse’s time expenditure, we also fitted a linear regression with restraint use as a binary independent variable. Binary outcomes were assessed accordingly by logistic regression. All models were fitted with robust standard errors. Analyses were performed with Stata version 15.2 (Stata Corp., College Station, TX, USA).

## 3. Results

### 3.1. Patient Cohort

**Figure 1:**
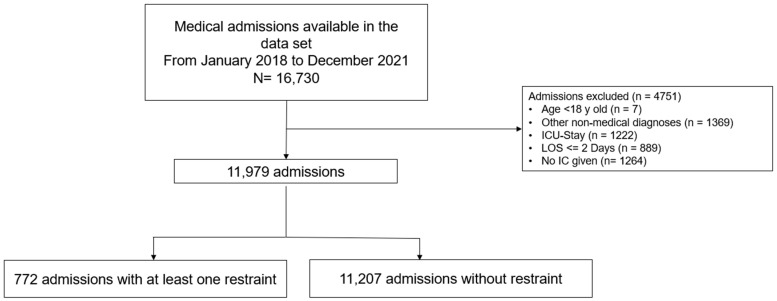
Flow chart of patient selection process. Abbreviation: LOS, length of stay; IC, informed consent; ICU, intensive care unit.

From January 2018 to December 2021, we reviewed 16,730 admissions of which 4751 met our exclusion criteria (see Figure 1). Out of 11,979 admissions, 772 (1.7%) experienced at least one physical restraint. The mean age was 78 years (±12), and 41.5% were women. The most frequent diagnoses in restrained patients were diseases of the circulatory system (22.0%), followed by diseases of the respiratory system (16.1%), and neoplasms (16.1%) (see Table 1). To further aid the readers’ interpretation, we added tables depicting the amount of missing information in the dataset (see Supplementary Tables S1 and S2), as we performed a complete case analysis only.

The most frequently used type of restraint was sensor mats (73.0%, n = 666), followed by blanket restrictions (14.5%, n = 132), bedrails (8.8%, n = 80) and any type of belt (3.7%, n = 34). 

The duration of a restraint normalized to the total number of restraints per patient was 42 h (±57). Bedrails had the longest duration with 58 h (±102), followed by sensor mats with 42 h (±51), blanket restrictions with 25 h (±23) and belts with 18 h (±21) (see Table 2). 

### 3.2. Association of Physical Restraints and Outcome

LOS was higher among patients with physical restraints with a median of 8 days (IQR 5–14), while patients without restraints had a median LOS of 5 days (IQR 3–9) (see Table 1). Nurses’ time expenditure per patient was performed by registered nurses and nurse assistants. Nurses spent a median of 309 h (IQR 242–402) in patients with restraints and 182 h (IQR 136–243) in patients without restraints. Falls occurred in 22.5% (n = 174) of restrained patients and in 2.7% (n = 300) of non-restrained patients. Only a minority of falls happened after the application of a restraint in 6.0% of patients (n = 46). However, there was a large spread in the timing between the use of restraint and occurrence of a fall (−19 h ± 161) (see Table 2). In-hospital death occurred in 13.3% (n = 103) of patients with physical restraints and 5.1 % (n = 577) of patients without physical restraints. Restraint patients also had a higher DOS (3.3) than those not restrained (0.55). 

After controlling for confounders by regression analysis, the utilization of physical restraint still had a significant impact on all the outcomes. For instance, baseline LOS was 4.28 days but was increased by an additional 2.31 days when restraining methods had to be applied to a patient. Additionally, baseline odds for a fall were very low at 0.005 but increased 8.85 times in the case of a restraining measure (see Table 3).

### 3.3. Indication for the Use of Restraint and Medical Therapy

The main reason for the use of restraint was delirium (64.6%, n = 137), followed by fall prevention (26.4 %, n = 56) and preventing aggressive behavior (3.8 %, n = 8). The use of benzodiazepines and antipsychotics tendentially increased during the hospital stay, and usage persisted after the stop of restraints (see Supplementary Table S3).

### 3.4. Trends over Time

**Figure 2:**
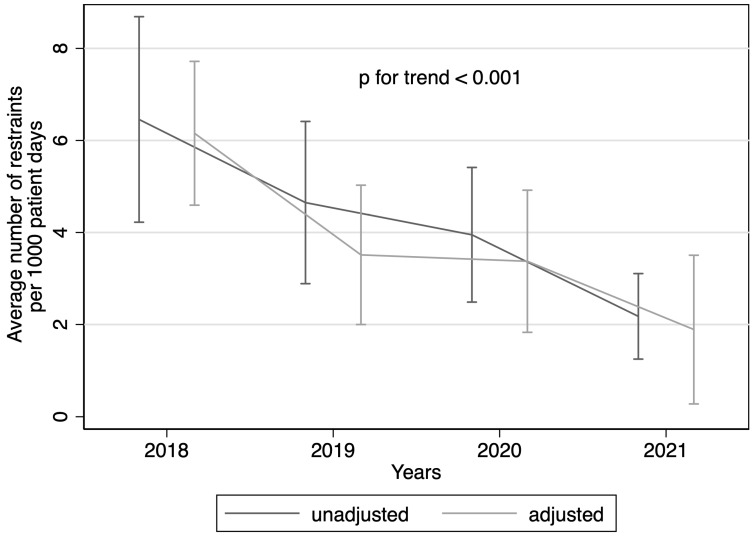
Use of restraints over the years. Use of restraints over the years fitted by unadjusted linear regression and adjusted by admission year, age, gender, main diagnosis, ward, insurance class, and Elixhauser index. y-axis with average number of restraints per 1000 patient days.

**Figure 3:**
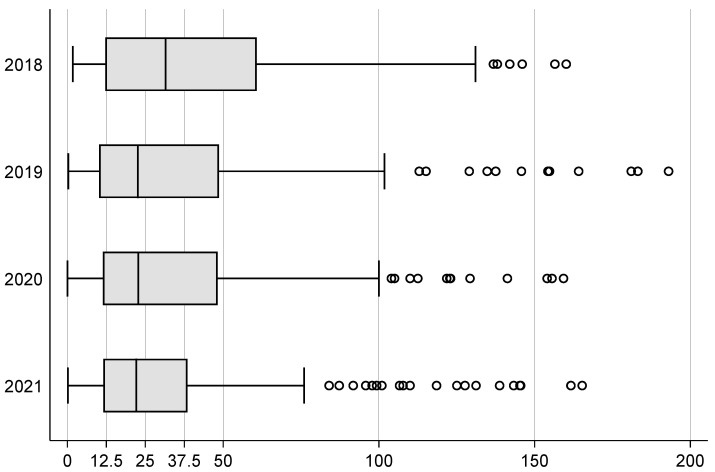
Boxplot of restraint duration in hours per total number of restraints. Legend: vertical bar in box denotes median, and edges of rectangles the 25th and 75th percentiles, whiskers extend to include all data points within 1.5 times the interquartile range, circles denote outside values. Note: Fourteen outliers between 200 h and 807 h were omitted for better scalability.

**Figure 4:**
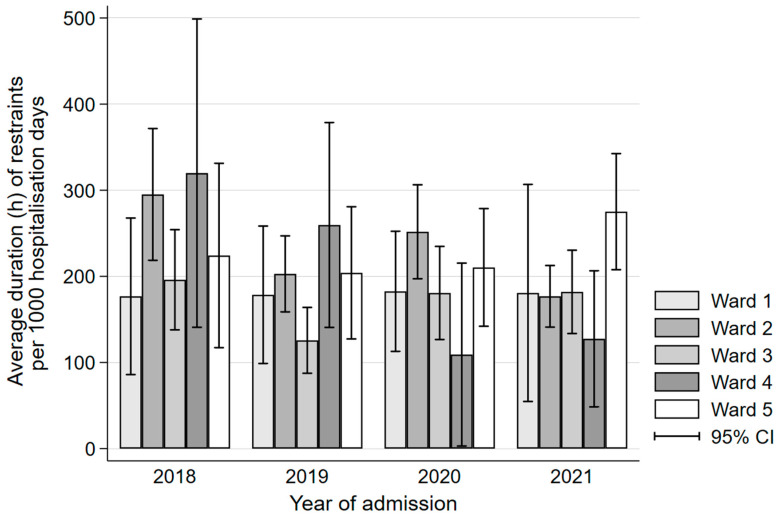
Bar chart of average duration in hours of restraint per year and ward. Legend: CI, confidence interval.

The use of restraints decreased significantly over the years (see Figure 2) while the average duration of restraint adjusted per 1000 patient days also declined (see Figure 3). There were no consistent differences in restraint use between wards in our clinic (see Figure 4).

## 4. Discussion

In our study, 6.4% out of 11,979 admissions received at least one physical restraint. Restrained patients were more severely ill, were more likely to be male, and older compared to non-restrained patients. The average restrained patient resulted in 309 h in nurses’ time expenditure, compared to 182 h for a non-restrained patient. LOS was 5 days in non-restrained patients, compared to 8 days in restrained patients. 22.5 % of restrained patients experienced a fall compared to 2.7% of non-restrained patients. Also, in-hospital death wase more frequent in restrained patients with 13.3% versus 5.1%. We also saw a decrease in the use of restraints over time, but no differences between wards. We assume that the statistically significant decrease in physical restraints over the time is due to the more liberal use of one-to-one supervision in our institution, however we do not have data to underpin this assumption.

Similar to other studies we have shown that physical restraint use might lead to several negative outcomes including increased risks of falls, aggressive behavior, a decline in physical functioning, and psychiatric comorbidities [2,14,15]. 

Our study corroborates that the vulnerable group of elderly and severely ill patients is mostly affected by restraint use.

### 4.1. LOS and Nurses’ Time Expenditure

Patients with physical restraints had a substantially longer LOS compared to non-restrained patients which is consistent with previous studies. Our average LOS for restraint patients was 8 days, which is below the 9 days to 21.1 days reported by others [6,8,9].

Restrained patients generate a large impact on hospital resources; in particular, nurses have to dedicate a lot, leaving them with less time for other tasks. These differences persisted despite adjustment for confounders providing evidence for a high-need patient group.

A detailed evaluation of a restraint’s necessity can help in minimizing both resources of personnel and making a stay for patients safer by preventing adverse events of restraint use, as patients often experience anger, discomfort, resistance, and fear in response to a physical restraint [16]. Providing patients with activities, or directly involving relatives could help minimizing or even avoiding the use of restraints [17]. Multidisciplinary decision making, adequate management of underlying co-precipitant factors such as sleep dysregulation, management of pain, adequate use of pharmacological therapy, and early mobilization alongside strengthening exercises are proven measures to minimize the use of restraints [18].

### 4.2. Comparing Fall and Type of Restraints

We observed that restraint use was mostly implemented before a fall. Use of physical restraints to prevent fall and ensuring patient’s safety is one of the most widespread indications [11]. Our study showed that 22.5% of restrained patients suffered a fall, which, on average, occurred 19 h before the restraint started. Because of the large heterogeneity in the data, we could not further delineate the associations between restraint use and a consecutive fall in a hierarchical regression model. While many restraints were started after a first fall, restraints do not seem to be effective in preventing a consecutive fall. 

This is in line with current evidence implying that restraints do not appear to be effective in reducing falls or injuries among adults in acute care hospitals [19,20]. It is even plausible that physical restraints paradoxically increase the risk of falls, as patients are more agitated, and attempting to escape their restraints may become entangled and fall [21]. In addition, the use of restraint may result in later mobilization another factor known to maintain a delirium [19,22].

While bedrails are the most common type of restraint in North America [23] and Germany [5], sensor mats were the most widely used type in our setting. We attribute this finding to the fact that regular, pre-installed, low raise bed rails are not considered a restraint, but only additionally installed high raise bed rails. 

### 4.3. Differences between Wards of the Same Clinic

A secondary analysis of a cross-sectional study in Germany showed that the prevalence of restraints was 9.3% in a sample of 2827 in-hospital patients and restraints use was more prevalent in women [24]. In contrast to our results, they observed a higher restraint rate on medical wards (12.5%, n = 116), and an even higher rate on the geriatric ward (25.6%, n = 30). However, comparability is difficult, as the patients in wards at our clinic are heterogeneous and polymorbid. 

A cross-sectional study in 55 Swiss hospitals found an average prevalence rate of physical restraints of 10.2% in 2021. However, large discrepancies were noted with 40% of all hospitals either using restraints significantly more or less often than the average [25]. The authors concluded that the use of physical restraints should be used as a quality indicator for hospitals. In our view, this call is questionable because of largely varying populations across clinics and hospitals. As a prerequisite, hospitals should be compared to one another clinic wise ensuring comparable patient risk groups and preventing mixing of differing patient populations.

### 4.4. Strengths and Limitations

As a single centered, retrospective cohort study, our results are not easily generalizable. Furthermore, we provide a descriptive overview and can thus draw no conclusions on causality between restraints and clinical outcomes. Additionally, we have a rather small sample for rare events, and no long-term follow-up outside an admission (e.g., 1 year mortality). In addition, our data warehouse currently only hosts up to five years of data, limiting our conclusions to a rather short time span. 

Our study has several strengths. We focused on the admission at medical wards with specific types of restraints and the chronological relation between the use of restraints and fall. In contrast to previous studies, we provide insight at a ward level and trends over the last four years. Further, we described nurses‘ time expenditure hours as a measure of work burden. Additionally, we provided information on the use of pharmacological therapy, and the classification of restraints’ indications.

## 5. Conclusions 

In conclusion, these data suggest that a relatively low rate of approximately 6% of medical patients experienced a restraint with a declining trend over time and no difference between the medical wards.

Our work points out the current challenges in nursing, that is economical pressure to reduce LOS combined with an increased number of elderly and severely ill in-hospital patients at high risk for delirium. In this challenging environment, nurses need to critically evaluate physical restraints as adverse effects from restraints are very common. We assume that patients with delirium are frequently subjected to physical restraints and specifically, we have shown that restraint use does not reduce consecutive falls. Hence, health care professionals should only apply restraints in situations where there is good evidence for a beneficial effect. As such, quality improvement studies should be conducted in various health care settings to find the best solutions for local problems. Additionally, restraints need to be used as shortly as possible, assessed at least once per shift, and be substituted by the least invasive measure such as one-to-one supervision whenever possible. Future studies should critically investigate the crucial relation between unfavorable patient-to-nurse ratios, especially as economical pressure will likely lead to reduced staffing. We need to better understand the optimal management of patients at high risk of a restraint to reduce the overall use of restraints and prevent their harmful complications. 

## Figures and Tables

**Table 1:** Baseline.

Factor	Overall	Without Restraints	With Restraints	*p*-Value ^¶^
	n = 11,979 (100%)	n = 11,207 (93.6%)	n = 772 (6.4%)	
Age, years	68 (±16)	68 (±16)	78 (±12)	<0.001
Female gender	5257 (43.9%)	4937 (44.1%)	320 (41.5%)	0.16
Elixhauser CMI	9.1 (±8.5)	8.8 (±8.4)	12.6 (±8.8)	<0.001
DOS points	0.8 (±1.7)	0.6 (±1.3)	3.3 (±2.3)	<0.001
DOS compliance *	972 (92.2%)	762 (91.9%)	210 (93.3%)	0.48
Major disease (ICD-10 Code)				
Diseases of the circulatory system	3339 (27.9%)	3169 (28.3%)	170 (22.0%)	
Diseases of the respiratory system	1861 (15.5%)	1737 (15.5%)	124 (16.1%)	
Neoplasms	1759 (14.7%)	1635 (14.6%)	124 (16.1%)	
Diseases of the digestive system	1245 (10.4%)	1178 (10.5%)	67 (8.7%)	
Certain infectious and parasitic diseases	1026 (8.6%)	946 (8.4%)	80 (10.4%)	
Diseases of the nervous system	566 (4.7%)	508 (4.5%)	58 (7.5%)	
Diseases of the genitourinary system	524 (4.4%)	482 (4.3%)	42 (5.4%)	
Disease of connective tissue	534 (4.5%)	519 (4.6%)	15 (1.9%)	
Endocrine, nutritional and metabolic diseases	343 (2.9%)	324 (2.9%)	19 (2.5%)	
Mental and behavioral disorders	219 (1.8%)	165 (1.5%)	54 (7.0%)	
Others	563 (4.7%)	544 (4.9%)	19 (2.5%)	<0.001
Place of discharge				
Outpatient care	8662 (72.3%)	8340 (74.4%)	322 (41.7%)	
Rehabilitation facility	2637 (22.0%)	2290 (20.4%)	347 (44.9%)	
Year of admission				
2018	2964 (24.7%)	2815 (25.1%)	149 (19.3%)	0.004
2019	3154 (26.3%)	2943 (26.3%)	211 (27.3%)	
2020	3034 (25.3%)	2823 (25.2%)	211 (27.3%)	
2021	2827 (23.6%)	2626 (23.4%)	201 (26.0%)	
LOS, days	6 (4–9)	5 (3–9)	8 (5–14)	<0.001
Nurses’ time expenditure, hours per admission	187 (13–255)	182 (136–243)	309 (242–402)	<0.001
In-hospital death	680 (5.7%)	577 (5.1%)	103 (13.3%)	<0.001

Data are presented as median (IQR) or mean (±SD) for continuous measures, and n (%) for categorical measures. Abbreviations.: DOS, Delirium Observation Scale (range 0–13 points); LOS, length of stay; ICD-10, International Classification of Diseases 10th Revision; CMI, comorbidity index. * Per internal protocol, nurses screened patients older than 65 years once per shift (i.e., three times per day) for the first three days of admission for the risk of delirium using the DOS. ^¶^ Comparison between “Without restraints” and “With restraints” columns only, continuous variables were compared by Wilcoxon rank-sum test, and categorical and binary variables by Pearson’s chi-squared test.

**Table 2:** Falls and outcomes among different types of restraints.

	Without Restraints	With Restraints	Blanket Restrictions	Belts	Bedrails	Sensor Mats
	n = 11,207 (98.3%)	n = 772 (1.7%)	n = 132 (17.1%)	n = 34 (4.4%)	n = 80 (10.4%)	n = 666 (86.3%)
Female gender	4937 (44.1%)	320 (41.5%)	54 (40.9%)	6 (18%)	39 (49%)	280 (42.0%)
DOS points	0.6 (±1.3)	3.3 (±2.3)	4.6 (±2.5)	4.3 (±2.5)	4.6 (±2.4)	3.1 (±2.1)
DOS compliance	762 (91.9%)	210 (93.3%)	35 (95%)	5 (71%)	17 (100%)	188 (93.5%)
Year of admission						
2018	2815 (25.1%)	149 (19.3%)	32 (24.2%)	14 (41%)	27 (34%)	112 (16.8%)
2019	2943 (26.3%)	211 (27.3%)	45 (34.1%)	10 (29%)	18 (23%)	185 (27.8%)
2020	2823 (25.2%)	211 (27.3%)	33 (25.0%)	6 (18%)	21 (26%)	185 (27.8%)
2021	2626 (23.4%)	201 (26%)	22 (16.7%)	4 (12%)	14 (18%)	184 (27.6%)
Place of discharges						
Outpatient care	8340 (74.4%)	322 (41.7%)	39 (29.5%)	8 (24%)	28 (35%)	286 (42.9%)
Rehabilitation facility	2290 (20.4%)	347 (44.9%)	70 (53.0%)	19 (56%)	34 (43%)	300 (45.0%)
Clinical outcomes						
LOS, days	5 (3–9)	8 (5–14)	8 (5.5–15)	13 (6–21)	11 (6–18)	8 (5–13)
Nurses’ time expenditure hours, hours per admission	182 (136–243)	309 (242–402)	341 (266–447)	344 (255–472)	376 (288–476)	304 (238–393)
Fall without restraint or after start of a restraint	300 (2.7%)	174 (22.5%)	35 (26.5%)	11 (32%)	29 (36%)	146 (21.9%)
Time difference between fall and restraint, hours *	N/A	−19 (±161)	−20 (±151)	−96 (±191)	−34 (±195)	−22 (±168)
Fall during first restraint use	N/A	46 (6.0%)	7 (5.3%)	0 (0%)	7 (9%)	39 (5.9%)
Restraint duration per total counts of restraints, hours	N/A	42 (±57)	25 (±23)	18 (±21)	58 (±102)	42 (±51)
In-hospital death	577 (5.1%)	103 (13.3%)	23 (17.4%)	7 (21%)	18 (23%)	80 (12.0%)

Data are presented as median (IQR) or mean (±SD) for continuous measures, and n (%) for categorical measures. Percentages are column wise. Figures do not add up to 100%, as patients could have had more than one physical restraint per admission. Abbreviation: DOS, Delirium Observation Screening Scale; LOS, length of stay; N/A, not applicable. * In the case of negative values, the fall happened before the start of a restraint; in the case of positive values, the fall happened during or after the start of a restraint.

**Table 3:** Outcomes multivariable adjusted by important clinical confounders.

Outcomes	Nurses’ Time Expenditure, Hours per Admission			LOS, Days			Fall			In-Hospital Death		
Variables	β	95% CI	*p*-Value	β	95% CI	*p*-Value	OR	95% CI	*p*-Value	OR	95% CI	*p*-Value
Physical restraint yes vs. no	96.29	87.12–105.45	<0.01	2.81	2.31–3.31	<0.01	8.85	6.85–11.45	<0.01	1.92	1.49–2.48	<0.01
Constant/Baseline	12.12	−0.65–24.89	0.06	4.28	3.58–4.98	<0.01	0.005	0.002–0.01	<0.01	0.001	0.001–0.003	<0.01
Age, years	1.67	1.52–1.82	<0.01	0.01	0.005–0.02	<0.01	1.02	1.01–1.03	<0.01	1.04	1.03–1.05	<0.01
Female, gender	7.18	2.75–11.61	<0.01	−0.19	−0.44–0.05	0.12	0.86	0.68–1.09	0.21	0.94	0.79–1.11	0.47
Elixhauser CMI	18.76	16.31–21.21	<0.01	1.57	1.43–1.70	<0.01	1.45	1.32–1.60	<0.01	1.37	1.27–1.47	<0.01
Year of admission												
2018	Ref.			Ref.			Ref.			Ref.		
2019	3.13	−2.85–9.10	0.31	0.02	−0.32–0.35	0.92	0.75	0.55–1.02	0.07	0.93	0.73–1.18	0.53
2020	30.66	24.67–36.65	<0.01	−0.30	−0.63–0.04	0.08	0.74	0.54–1.01	0.06	0.85	0.66–1.08	0.17
2021	30.15	23.70–36.61	<0.01	−0.003	−0.35–0.34	0.98	0.73	0.53–1.01	0.06	1.08	0.85–1.37	0.51
Major disease (ICD-10 Code)												
Diseases of the circulatory system	Ref.			Ref.			Ref.			Ref.		
Diseases of the respiratory system	75.18	69.10–81.27	<0.01	0.77	0.44–1.11	<0.01	1.22	0.87–1.72	0.25	1.58	1.21–2.05	<0.01
Neoplasms	52.31	45.78–58.85	<0.01	3.37	3.02–3.73	<0.01	2.12	1.56–2.88	<0.01	3.77	2.99–4.77	<0.01
Diseases of the digestive system	49.66	42.70–56.62	<0.01	0.49	0.11–0.87	0.01	0.84	0.54–1.30	0.43	0.77	0.53–1.13	0.19
Certain infectious and parasitic diseases	81.98	74.50–89.45	<0.01	1.19	0.78–1.59	<0.01	1.19	0.79–1.79	0.40	2.75	2.09–3.60	<0.01
Class of insurance	−13.00	−19.75–(−6.25)	<0.01	−0.37	−0.74–0.01	0.05	1.02	0.73–1.44	0.89	0.94	0.73–1.21	0.65
Medical Wards												
1	Ref.			Ref.			Ref.			Ref.		
2	23.52	16.33–30.71	<0.01	−0.31	−0.70–0.08	0.12	1.02	0.68–1.53	0.91	1.25	0.93–1.69	0.14
3	2.67	−4.62–9.96	0.47	0.35	−0.05–0.75	0.09	1.18	0.79–1.77	0.43	1.07	0.79–1.45	0.67
4	3.05	−5.98–12.08	0.51	0.76	0.27–1.24	<0.01	1.20	0.75–1.92	0.44	1.09	0.78–1.54	0.61
5	0.83	−7.266–8.92	0.84	0.35	−0.09–0.79	0.12	1.17	0.76–1.82	0.48	1.24	0.90–1.72	0.19

Abbr.: Ref., Reference; CI, confidence interval; OR, odds ratio; LOS, length of stay; ICD-10, International Classification of Diseases 10th Revision. Legend: Outcomes multivariable adjusted by important clinical confounders either by linear regression in case of LOS and Nurses’ time expenditure, or in case of fall and in-hospital death by logistic regression. Outcomes are multivariably adjusted for the above covariates including restraint use.

## Data Availability

According to Swiss laws, data sharing is restricted to the country.

## Supplementary Materials

The following supporting information can be downloaded at: https://www.mdpi.com/article/10.3390/geriatrics8010015/s1, Table S1: Depiction of missing data in Table 1. Table S2: Depiction of missing data in Table 2. Table S3: Indication for use of restraints and therapy with psychotropic medications.
