# Supplementary material for: Prevalence, Risk Factors and Outcomes Associated with Physical Restraint in Acute Medical Inpatients over 4 Years—A Retrospective Cohort Study
Source: Geriatrics (Basel). 2023 Jan 17;8(1):15. doi: 10.3390/geriatrics8010015 (PMC9957493; doi:10.3390/geriatrics8010015)
Supplement: Supplementary file 1 [file geriatrics-08-00015-s001.zip › geriatrics-2069479-supplementary.pdf]

Supplementary Appendix

Prevalence, Risk Factors and Outcomes Associated with Physical Restraint in Acute Medical Inpatients over 4 Years—A Retrospective Cohort Study

Umberto Spennato [1], Nathalie Lerjen [1,*], Jennifer Siegwart[1], Beat Mueller[1,2], Philipp Schuetz[1,2], Daniel Koch[1,¶], Tristan Struja[1,¶]

[1] Medical University Clinic, Kantonsspital Aarau, Aarau, Switzerland

[2] Medical Faculty of the University of Basel, Switzerland

* Equally contributing first authors

¶ Equally contributing last authors.

VERSION: December 27, 2022

Corresponding author and person to whom reprint requests should be addressed:

Umberto Spennato, MUDr.

Medical University, Kantonsspital Aarau

Tellstrasse

CH-5001 Aarau, Switzerland

umberto.spennato@ksa.ch (E-Mail)

Supplementary Appendix

Table S1: Depiction of missing data in table 1.

Table S2: Depiction of missing data in table 2.

Table S3: Indications for use of restraints and therapy with psychotropic medications.

Table S1: Missing Data from table 2.

Factor	Overall	Without restraints	With restraints	Overall (missing)	Without restraints (missing)	With restraints (missing)
	**N=11,979**	**N=11,207**	**N=772**			
Age, years	11,979	11,207	772	0	0	0
Gender	11,979	11,207	772	0	0	0
Elixhauser CMI	10,898	10,200	698	1,081	1,007	74
DOS points	4,512	4,073	439	7,467	7,134	333
Major disease (ICD-10 Code)	11,979	11,207	772	0	0	0
Place of discharge	11,979	11,207	772	0	0	0
Year of admission	11,979	11,207	772	0	0	0
LOS, days	11,979	11,207	772	0	0	0
Nurses' time expenditure, hours per admission	11,468	10,731	737	511	476	35
In-hospital death	11,979	11,207	772	0	0	0

Table S2: Depiction of missing data in table 2, part A available data.

	Without restraints	With restraints	Blanket restrictions	Belt	Bedrails	Sensor mats
	**N=11,207**	**N=772**	**N=132**	**N=34**	**N=80**	**N=666**
Gender	11,207	772	132	34	80	666
DOS points	4,073	439	63	13	38	391
Year of admission	11,207	772	132	34	80	666
Place of discharges	11,207	772	132	34	80	666
LOS, days	11,207	772	132	34	80	666
Nurses' time expenditure hours, hours per admission	10,731	737	128	32	76	634
Fall without restraint or after start of a restraint	11,207	772	132	34	80	666
Time difference between fall and restraint, hours*	0	174	35	11	29	146
Fall during first restraint use	11,207	772	132	34	80	666
Restraint duration per total counts of restraints, hours	0	772	132	34	80	666
In-hospital death	11,207	772	132	34	80	666

Table S2: Depiction of missing data in table 2, part B missing data.

	Without restraints (missing)	With restraints (missing)	Blanket restrictions (missing)	Belt (missing)	Bedrails (missing)	Sensor mats (missing)
	**N=11,207**	**N=772**	**N=132**	**N=34**	**N=80**	**N=666**
Gender	0	0	0	0	0	0
DOS points	7,467	333	69	21	42	275
Year of admission	0	0	0	0	0	0
Place of discharges	0	0	0	0	0	0
LOS, days	0	0	0	0	0	0
Nurses’ time expenditure hours, hours per admission	476	35	4	2	4	32
Fall without restraint or after start of a restraint	0	0	0	0	0	0
Time difference between fall and restraint, hours*	11,207	598	97	23	51	520
Fall during first restraint use	0	0	0	0	0	0
Restraint duration per total counts of restraints, hours	11,207	0	0	0	0	0
In-hospital death	0	0	0	0	0	0

Table S3: Indications for use of restraints and therapy with psychotropic medications.

Indications	Total	Blanket restriction	Belt	Bedrails	Sensor mats¶
	**N=772 (100%)**	**N=132 (17.1%)**	**N=34 (4.4%)**	**N=80 (10.4%)**	**N=666 (86.3%)**
Delirium	137 (64.6%)	99 (75.0%)	21 (61.0%%)	42 (52.0%)	N/A
(Preventing) Falls	56 (26.4%)	27 (20.5%)	7 (21.0%)	28 (35.0%)	N/A
(Preventing) Aggressive behavior	8 (3.8%)	4 (3.0%)	4 (12.0%)	1 (1.0%)	N/A
(Preventing) Wandering around	3 (1.4%)	2 (1.5%)	0 (0.0%)	2 (3.0%)	N/A
Other motive: Patient's request, Non-compliance with treatment, etc.	8 (3.8%)	0 (0.0%)	2 (6.0%)	7 (9.0%)	N/A
Benzodiazepines*					
No benzodiazepine	83 (39.2%)	51 (38.6%)	10 (29.0%)	32 (40.0%)	N/A
Before first restraint	20 (9.4%)	10 (7.6%)	3 (9.0%)	9 (1.0%)	N/A
During first restraint	36 (17.0%)	21 (15.9%)	7 (21.0%)	15 (19.0%)	N/A
After first restraint	73 (34.4%)	50 (37.9%)	14 (41.0%)	24 (30.0%)	N/A
Antipsychotics#					N/A
No antipsychotics	47 (22.2%)	26 (19.7%)	7 (21.0%)	19 (24.0%)	N/A
Before first restraint	15 (7.1%)	8 (6.1%)	3 (9.0%)	5 (6.0%)	N/A
During first restraint	41 (19.3%)	25 (18.9%)	6 (18.0%)	17 (21.0%)	N/A
After first restraint	109 (51.4%)	73 (55.3%)	18 (43.0%)	39 (49.0%)	N/A

Data are presented as median (IQR) for continuous measures, and n (%) for categorical measures. We manually extracted information on these restrictions, as the inclusion of the other restrictions occurred during the peer-review process, we do not have information for sensor mats.

Abbr.: N/A, not available

* The most frequently used Benzodiazepines were lorazepam, midazolam and zolpidem.

\# The most frequently used Antipsychotics were quetiapine, haloperidol and pipamperone.

¶ Sensor mats were included into the study during the peer-review process, why hand searching further data was not possible anymore.
